# Role of Molecular Orbital Energy Levels in OLED Performance

**DOI:** 10.1038/s41598-020-66946-2

**Published:** 2020-06-18

**Authors:** Rohit Ashok Kumar Yadav, Deepak Kumar Dubey, Sun-Zen Chen, Tzu-Wei Liang, Jwo-Huei Jou

**Affiliations:** 1 Department of Materials Science and Engineering, National Tsing Hua University, Hsinchu 30013, Taiwan, Republic of China; 2 Center for Nanotechnology, Materials Science, and Microsystems, National Tsing Hua University, Hsinchu 30013, Taiwan, Republic of China; 3 Global Science Instruments Co., New Taipei City 23144, Taiwan, Republic of China

**Keywords:** Materials science, Optics and photonics

## Abstract

Abundant molecules enable countless combinations of device architecture that might achieve the desirable high efficiency from organic light-emitting diodes (OLEDs). Due to the relatively high cost of OLED materials and facilities, simulation approaches have become a must in further advancing the field faster and saver. We have demonstrated here the use of state-of-art simulation approaches to investigate the effect of molecular orbital energy levels on the recombination of excitons in OLED devices. The devices studied are composed of 1,1-bis[(di-4-tolylamino)phenyl]cyclohexane (TAPC) as hole transporting material (HTM), 4,4′-Bis(9-carbazolyl)-1,1′-biphenyl (CBP) as host, 2,2',2”-(1,3,5-benzinetriyl)-tris(1-phenyl-1-H-benzimidazole) (TPBi) or bathophenanthroline (Bphen) as electron transporting materials. The outcomes reveal that exciton recombination highly sensitive to the energy-level alignment, injection barriers, and charge mobilities. A low energy-barrier (<0.4 eV) between the layers is the key to yield high recombination. The lowest unoccupied molecular orbital (LUMO) levels of the organic layers have played a more pivotal role in governing the recombination dynamics than the highest occupied molecular orbital (HOMO) level do. Furthermore, the Bphen based device shows high exciton recombination across the emissive layer, which is >10^6^ times greater than that in the TPBi based device. The high carrier mobility of Bphen whose electron mobility is 5.2 × 10^−4^ cm^2^ V^−1^ s^−1^ may lead to low charge accumulation and hence high exciton dynamics. The current study has successfully projected an in-depth analysis on the suitable energy-level alignments, which would further help to streamline future endeavours in developing efficient organic compounds and designing devices with superior performance.

## Introduction

Organic light-emitting diodes (OLEDs) have drawn enormous attention due to their outstanding performance in high-quality full-color display and solid-state lighting applications^1–3^. Numerous OLED displays have been introduced successfully to the global market, and lighting products are developing quickly. Whilst, device performance and lifetime are always potential reliability concern before they can be adopted more extensively, especially for lighting purpose^4–7^. Numerous factors have been identified that significantly contribute to the overall performance of the OLEDs. Studies suggested that device architecture and material characteristics are needed to be considered before fabrication in the pursuit of high device efficiency and good color quality^8,9^.

In order to develop products with energy-saving and long-lasting properties, high-efficiency OLEDs are highly essential^10–12^. The factors influencing the efficiency and other performance include carrier-injection^13–15^, charge mobility^16,17^ and device architecture^18–21^. Specifically, for high-efficiency OLEDs, a multilayer device architecture that consists of a hole-injection/transport layer (HIL/HTL), emissive layer (EML), and electron-injection/transport layer (EIL/ETL) is used. This provides feasibility to employ materials with appropriate molecular orbital energy levels and charge carrier mobility. One of the important criteria in the selection of materials is to have a minimum carrier-injection barrier across the layers in OLEDs^22–25^.

The carrier-injection plays an important role because the imbalance of charges often accounts for the low efficiency observed in typical devices. The devices with a high carrier-injection barrier depreciate the device efficiency due to an unwanted accumulation of carriers before reaching the EML. To overcome such losses in devices, a step-wise energy transport architecture is used. Another approach to improve the device efficiency is to insert a hole-modulating layer (HML) between HTL/EML or EML/ETL to obtain balanced carrier-injection into the emissive layer. The step-wise energy transfer renders superiority in OLED devices because of balanced carrier injection and effective energy transfer, which leads to maximum exciton recombination and high device performance. From materials perspective, an electron-rich transporting material with matching energy levels enables a minimal the carrier-injection barrier, hence improving the device efficiency^26–30^.

To shed some light on the roles of carrier-injection and mobility, Adachi *et al*. proposed a key design feature to achieve high device durability by fixing a small energy barrier at the interfaces^31^. In 2008, Hou’s group successfully synthesized a band gap-tunable material that can tune HOMO/LUMO effectively to provide high-triplet energy and good transporting ability^32^. In 2011, Jou *et al*. proposed double mixed-host device architecture to disperse the injected carriers into different recombination zones to enhance lifetime and efficiency^33^. In 2013, Chang *et al*. proposed a mechanism to align the energy level between the HTL and EML to effectively mitigate operational voltages. Confining the carriers, especially at high voltage, over an appropriate layer becomes crucial for the realization of reduced efficiency roll-off^34^. More recently, Jou *et al*. demonstrated a simulation-experiment study to prove the key importance of transporting materials, charge mobility, and device architecture in recombination rate and device performance^22^. A simulation model assists us to understand and delineate the physical processes within the devices and to prophesy of the behaviors of the next devices in advanced. However, there were very few reports on real-time simulation of OLED devices that could explain the role of molecular energy levels and injection barriers on OLED efficiency^35,36^.

In the present work, we have demonstrated a comprehensive drift-diffusion simulation approach to investigate the effect of molecular orbital on the recombination of exciton in OLED devices. The drift-diffusion formulation incorporated in SETFOS provided a vivid picture of the recombination profile of multi-layered OLED devices with respect to change in optoelectronic properties. The outcomes reveal that exciton recombination is highly sensitive to the energy-level alignment, injection barriers, and charge mobilities. A low energy-barrier (<0.4 eV) between the layers is the key to yield high recombination. The lowest unoccupied molecular orbital (LUMO) levels of the organic layers play a more pivotal role in governing the recombination dynamics than the highest occupied molecular orbital (HOMO) levels.

## Drift-diffusion model

To analyze the effective alignment of the frontier molecular orbital energies on the underlying physics and performance of OLEDs, a commercially available numerical simulator, SETFOS (Semiconducting Thin Film Optics Simulation Software), is used. The SETFOS is dealing with state-of-the-art mobility models: Poole Frenkel, EGDM/ECDM, constant mobility, which are modifying from the basic drift-diffusion model^37^. Poisson and Continuity equations based simulation model is used to investigate the electric field distribution and time-invariant as illustrated by the equations.

1 $$\frac{dE}{dx}=\frac{q}{\varepsilon {\varepsilon }_{0}}(p-n)$$

2 $$\frac{dn}{dt}=\frac{1}{q}\frac{d{J}_{n}}{dx}+G-R$$

3 $$\frac{dp}{dt}=-\frac{1}{q}\frac{d{J}_{p}}{dx}+G-R$$

Where, *E* is the electric field across the device, *q* is the elementary charge, *ɛ*_0_ is the dielectric permittivity of the organic materials, *ɛ*_*r*_ is the dielectric constant, *p* and *n* are the hole and electron density respectively, *J*_*n*_ is the electron current density, *R* is the recombination rate, and *G* represents the generation rate. The carrier density distribution across the device can be integrated by resolving the drift and diffusion equations via EGDM/ECDM. Furthermore, the conventional electric field dependent Poole-Frenkel mobility model for carrier mobility calculation in the semiconductor devices is represented by the equations.

4 $$R=\frac{q}{\varepsilon {\varepsilon }_{0}}({\mu }_{n}+{\mu }_{p})$$

5 $$u={\mu }_{0}\,exp\left\{-{\left(\frac{2\sigma }{3{k}_{B}T}\right)}^{2}+C\left[{\left(\frac{2\sigma }{3{k}_{B}T}\right)}^{2}-{\sum }^{2}\right]\sqrt{E}\right\}$$

6 $$\mu (E)={\mu }_{0}exp(\gamma \sqrt{E})$$

Here, *µ*_*p*_ and *µ*_*n*_ are the hole and electron mobility respectively, *D*_*n*_ the diffusion constant, *µ*_*n*_*(E)* is the charge carrier mobility under the electric field, *µ*_0_ is the mobility under zero electric field, *γ* is the Poole-Frenkel field dependent factor. The recombination rate R, the generation rate G does not depend on the carrier concentration; it is just a reflection on the thermal energy contained in the system and therefore pretty much constant. The Tapping rate, in a steady-state condition, the net Trapping rate of electrons should match the net recombination rate for holes, ${R}_{nt}-{G}_{n}={R}_{pt}-{G}_{p}$ and leads to the SRH expression for the trap-assisted recombination: $=\frac{np}{{\tau }_{n}(p+\,{p}_{t})\,+\,{\tau }_{p}(n+\,{n}_{t})}$. ^38–45^.

## Device structure and simulation parameters

**Figure 1:**
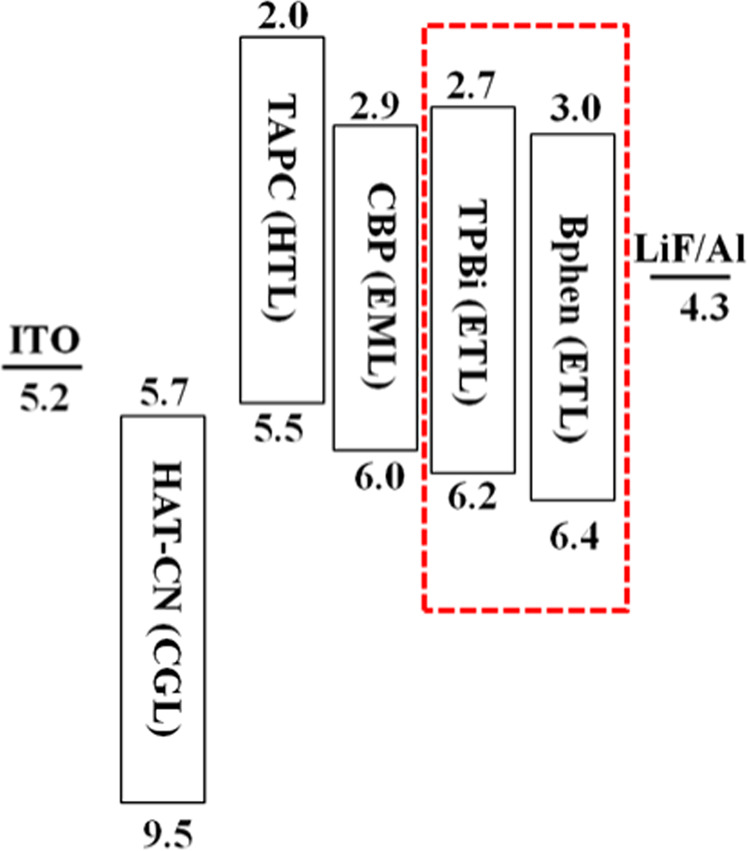
Schematic energy levels diagram of the studied OLED devices with the TPBi and Bphen as electron transporting materials.

**Figure 2:**
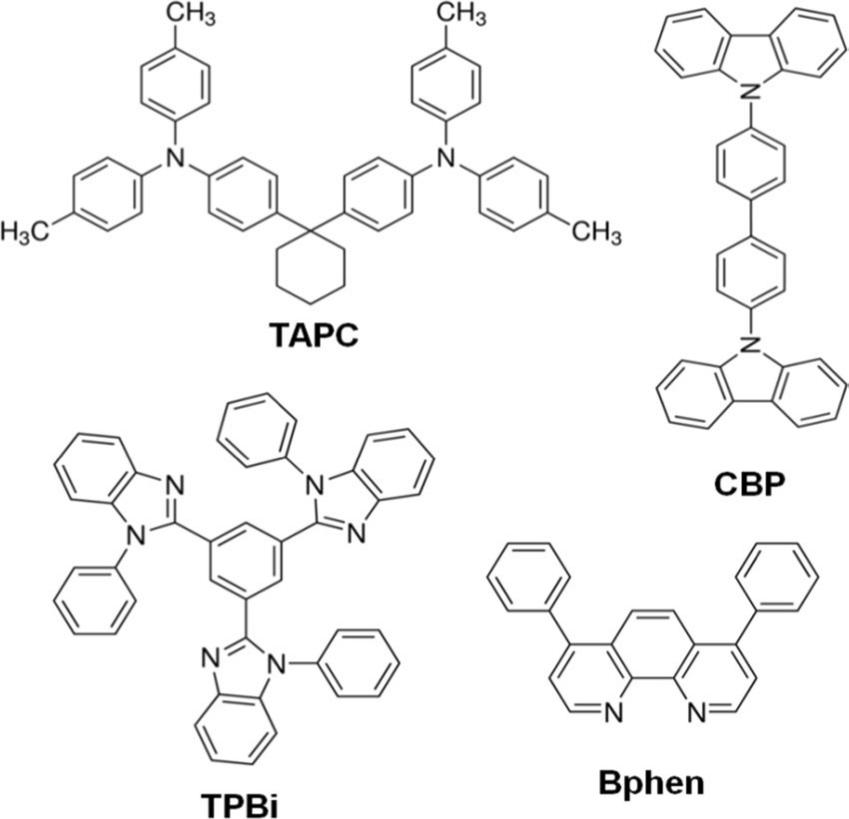
Molecular structures of the hole transport material (TAPC), electron transport materials (TPBi and Bphen) and host (CBP) used in this study.

**Figure 3:**
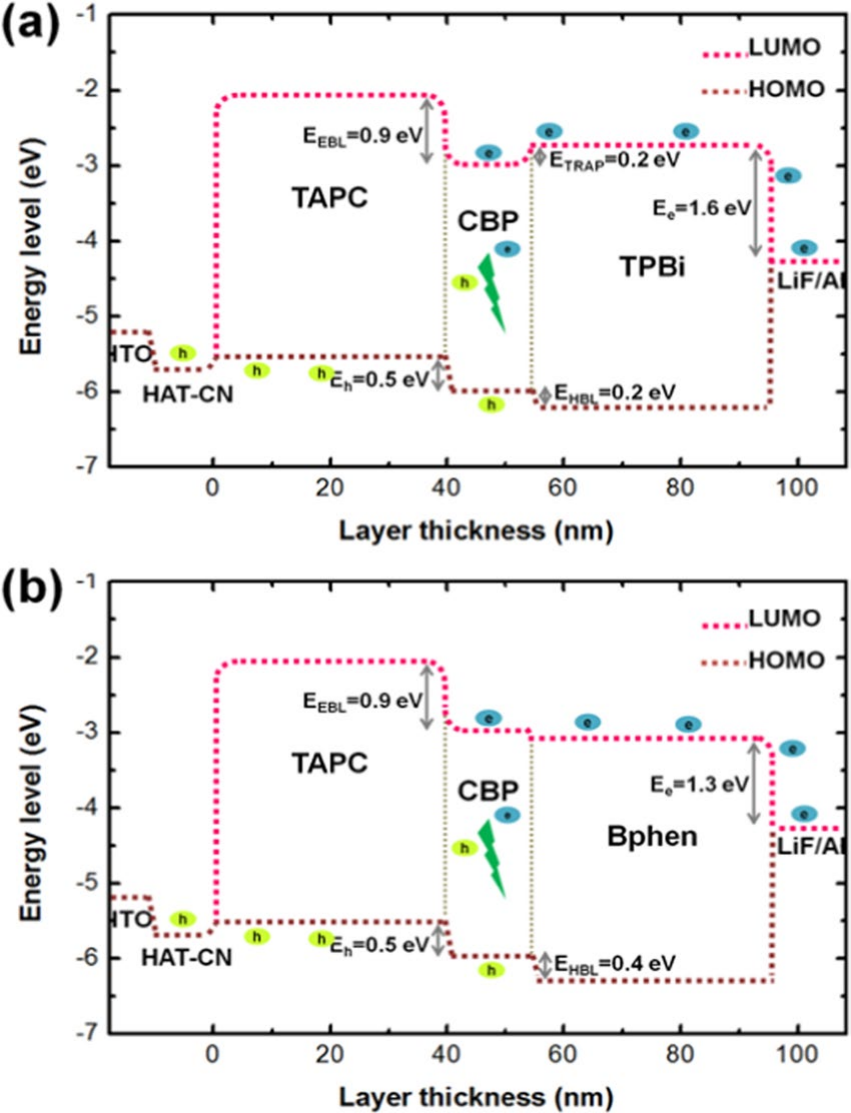
The energy-level alignment diagram of (**a**) the TPBi-composing control device and (**b**) the Bphen-composing one with the LUMO and HOMO energies originating at the interface of the organic layers and the Fermi levels of the electrode materials.

Table 1 summarizes the values input in the simulation for the studied OLED devices that include the HOMO, LUMO, hole mobility, and electron mobility. Figure 1 displays the studied OLED device architecture and their corresponding energy levels. The device structure consisted of a 125 nm indium tin oxide (ITO) as an anode layer, a 3 nm 1,4,5,8,9,11-hexaazatriphenylenehexacarbonitrile (HAT-CN) as hole injection layer (HIL), a 40 nm layer of 4,4ʹcyclohexylidenebis-N,N-bis(4methylphenyl)benzene-amin (TAPC) as hole transporting layer (HTL), a 15 nm single emissive layer (EML) with 15 wt% tris[2-phenylpyridinato-C2,N]iridium(III) Ir(ppy)_3_ doped in 4,4′-bis(N-carbazolyl)-1,1′-biphenyl (CBP) host, a 40 nm layer of 1,3,5-tris(N-phenyl-benzimidazol-2-yl)benzene (TPBi) as electron transport layer (ETL), a 1 nm lithium fluoride (LiF) as electron injection layer (EIL), and a 100 nm aluminum as cathode. Besides TPBi, another molecule, 4,4-bis(carbazol-9-yl)biphenyl (Bphen) was studied for the comparison. All the simulations were performed at a fixed operating voltage (3 V). Figure 2 illustrates the molecular structures of the materials used in this study. Figure 3 displays the energy level alignment diagram of the studied devices by showing the LUMO and HOMO energies originating at the interfaces of the organic layers and the Fermi levels of the electrode materials.

**Table 1:** The values input in the simulation for the studied OLED devices that include the HOMO, LUMO, hole mobility, and electron mobility^46,47^.

Material	HOMO (eV)	LUMO (eV)	µ_h_ (cm^2^ V^−1^s^−1^)	µ_e_ (cm^2^V^−1^s^−1^)	Function/ Thickness(nm)
TAPC	5.5	2.0	1.0 × 10^−2^	—	HTL/40
CBP	6.0	2.9	2.0 × 10^−3^	3.0 × 10^−4^	Host/15
TPBi	6.2	2.7	—	3.3 × 10^−5^	ETL/40
Bphen	6.4	3.0	—	5.2 × 10^−4^	ETL/40
Ir(ppy)_3_	5.7	2.9	—	—	Emitter/15
HAT-CN	9.5	5.7	—	—	HIL/3

Applied simulation operation voltage = 3.0 V.

Operation temperature = 298 K, Device area = 0.9 cm^2^, Dielectric constant = 3.5.

## Results and discussion

### Carrier recombination distribution of the TPBi based devices

**Figure 4:**
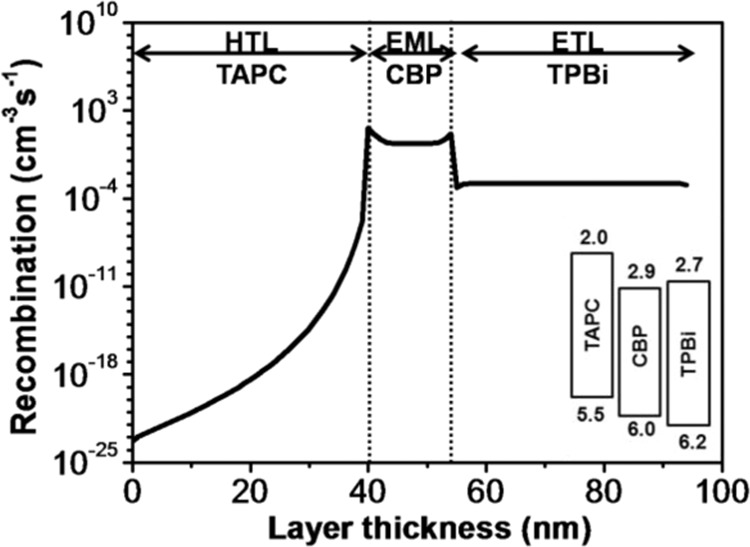
Recombination profile across the layers of the TPBi-ETL based control device.

Figure 4 shows the recombination profile of the TPBi composing control device. The simulation outcome shows that the recombination occurred in the EML is higher than both the adjacent layers, i.e., HTL and ETL. Therefore, the total radiative recombination within the EML is comparatively much higher than that of the HTL and ETL, which might be leading to highly efficient and long lifespan OLED device. It is notable that two radiative recombination peaks are locating at the interfaces within the EML due to the cascade traits of the molecular-orbital alignments at the interfaces. This cascade structure may help the capture and trap of carriers at the interface providing efficient sites for the recombination of the injected hole-electron pairs and resultant electroluminescence. At the HTL/EML interface, the comparatively low HOMO energy barrier (0.5 eV) still can effectively transfer the holes from the TAPC to the CBP. Moreover, the even high LUMO barrier (0.9 eV) blocks electrons more effectively. As result, the blocked electrons can quickly meet with the transferred holes forming the excitons near the HTL/EML interface. It is why the device shows the highest recombination peak at the HTL/EML interface. As holes are the major carriers of the studied devices, the available mobility of the holes are more substantial than that of electrons. It means that only parts of the holes are reacted with the blocked electrons at the HTL/EML, portions of the holes can overflow through the EML. So, some of the over-flowed holes can meet with the electrons which injected from the ETL and form excitons in the center region of the EML. Because there is no energy level misalignment within the EML, the resultant total radiative recombination shows a plateau in the center region. At the EML/ETL interface, the unreacted holes can be blocked and accumulated by the 0.2 eV HOMO energy gap and meet with the electrons that injected from the cathode and ETL. That is why the device shows a second highest recombination peak at the EML/ETL interface.

#### Effect of hole transporting layer

**Figure 5:**
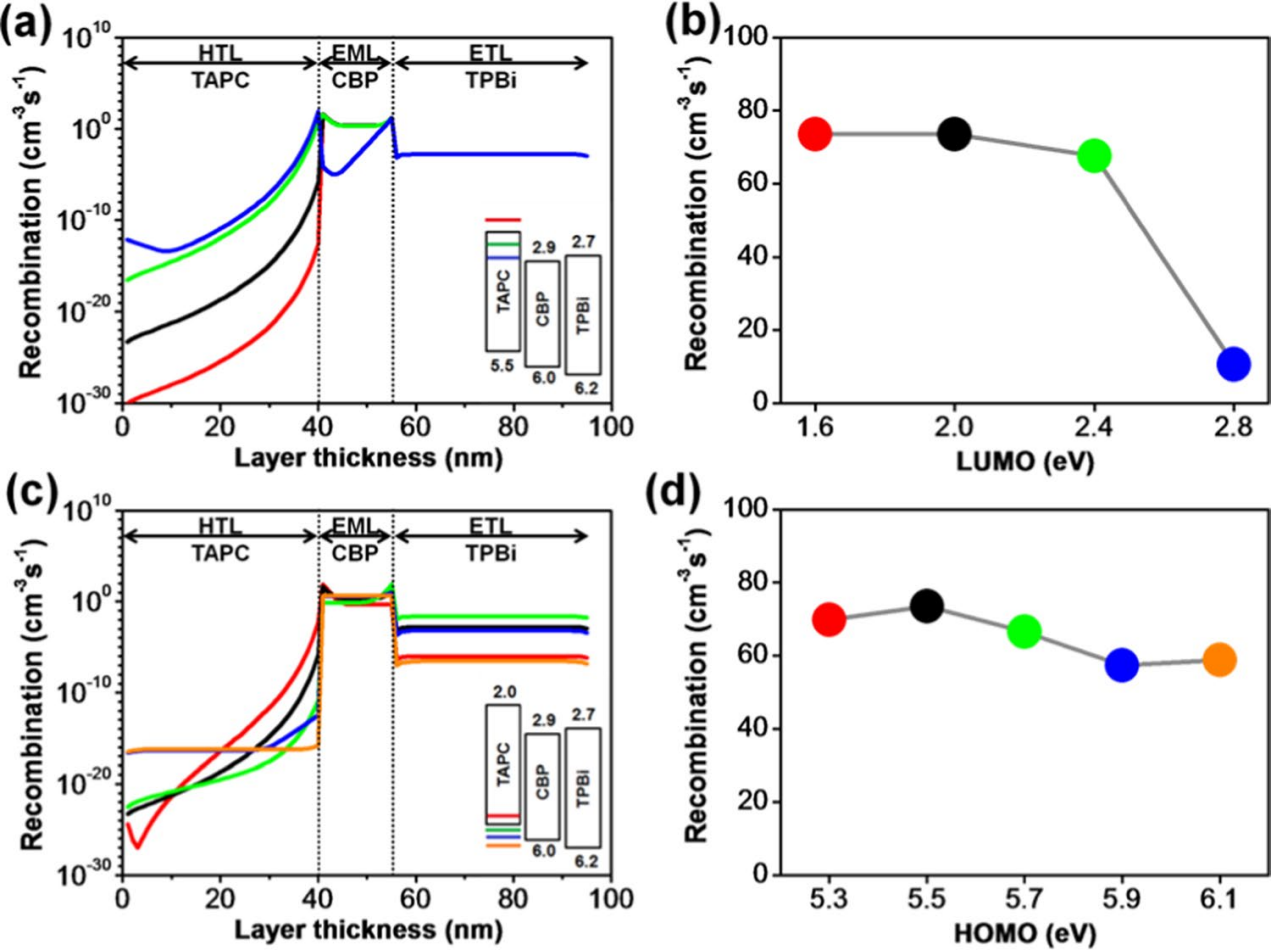
(**a**) Recombination distribution profiles in each organic layer corresponding to the simulated LUMO levels of TAPC, (**b**) effects of LUMO levels of TAPC on the total radiative recombination rates across the EML, (**c**) recombination distribution profiles in each organic layer corresponding to the simulated HOMO levels of TAPC, and (**d**) effects of HOMO levels of TAPC on the total radiative recombination rates across the EML of TPBi composing OLEDs.

Figure 5 reveals the effects of the molecular orbital energy levels of TAPC on the recombination profiles across the organic layers and interfaces. Figure 5a illustrates the influence of the LUMO levels of TAPC on the recombination rates across the device, and Fig. 5b shows the total radiative recombination rates within the EML with different LUMO levels. It seems that the total recombination rate across the HTL and EML is heavily influenced by the LUMO level. The total radiative recombination rate within the EML significantly decreases from 73.5 to 10.5 cm^−3^s^−1^, a decrement of 85%, as the LUMO level of TAPC changes from 1.6 to 2.8 eV. The reason can be attributed to the significant decrement of the electron blocking ability of the TAPC at the HTL/EML interface, i.e. from 1.3 to 0.1 eV. The injected electrons can accumulate at the HTL/EML interface, as the LUMO level of HTL is high enough to form an effective barrier. If it is not sufficiently high, the accumulated electrons may overflow from the EML to the HTL. In this case, the energy of the excess electrons is wasted as joule heating rather than exciton formation. This also interprets why the recombination profile within the HTL exponentially increases as the LUMO level changes from 1.6 to 2.8 eV resulting from the facility of the electron migration from the EML to HTL leading to the increase of the recombination rate in the HTL. While the recombination across the ETL illustrates a consistent profile for all LUMO levels of TAPC. It might attribute to that the holes are the major carriers and the available density of them are more than that of the electrons. In the cases of Fig. 5a,b, the HOMO levels of both EML and ETL are fixed, then a certain fraction of holes can move to the ETL resulting in a consistent recombination profile as shown in Fig. 5a.

Figure 5c illustrates the influence of the HOMO levels of TAPC on the recombination rates across the device and Fig. 5d shows the total radiative recombination rates within the EML with different HOMO levels. The total radiative recombination rate within the EML decreases from 73.5 to 66.5 cm^−3^s^−1^, a decrement of 9.5%, as the HOMO level changes from 5.5 to 5.7 eV. While the total recombination rate within the ETL is increased from 5.8 × 10^−2^ to 8.4 × 10^−1^ cm^−3^s^−1^, the reason behind this may be due to a massive inward migration of holes from the HTL to EML and then to ETL, as the energy barrier of the HTL/EML interface reduces from 0.5 to 0.3 eV. Resulting, imbalance of charge movements and accumulations of holes in the EML and ETL leads to the decrease of the recombination in the EML and the increase of that in the ETL. Moreover, by further modulating the HOMO value of the HTL from 5.7 to 6.1 eV, a 13% decrement in the total radiative recombination rate from 66.5 to 58.7 cm^−3^s^−1^ is observed across the EML. The total radiative recombination within the EML slightly declined as the HOMO level is modulated from 5.5 to 5.3 eV for increasing the energy barrier of the HTL/EML interface causing inefficient transfer of holes from the HTL to EML and the accumulation of the holes in the HTL.

#### Effect of host

Host materials are quite relevant to determine the overall performances of the optoelectronic devices since the hosts usually are the major constituent of the EML. Therefore, it is very crucial to select a host whose energy level aligns well with the adjacent layers, such as ETL and HTL, and the employed emitter resulting in an efficient energy transfer. An ideal host material should possess significantly proper molecular-orbital energy alignment, high triplet energy (E_T_), a bipolar charge transporting nature, and sufficient spectral overlap with the emitter^46^.

**Figure 6:**
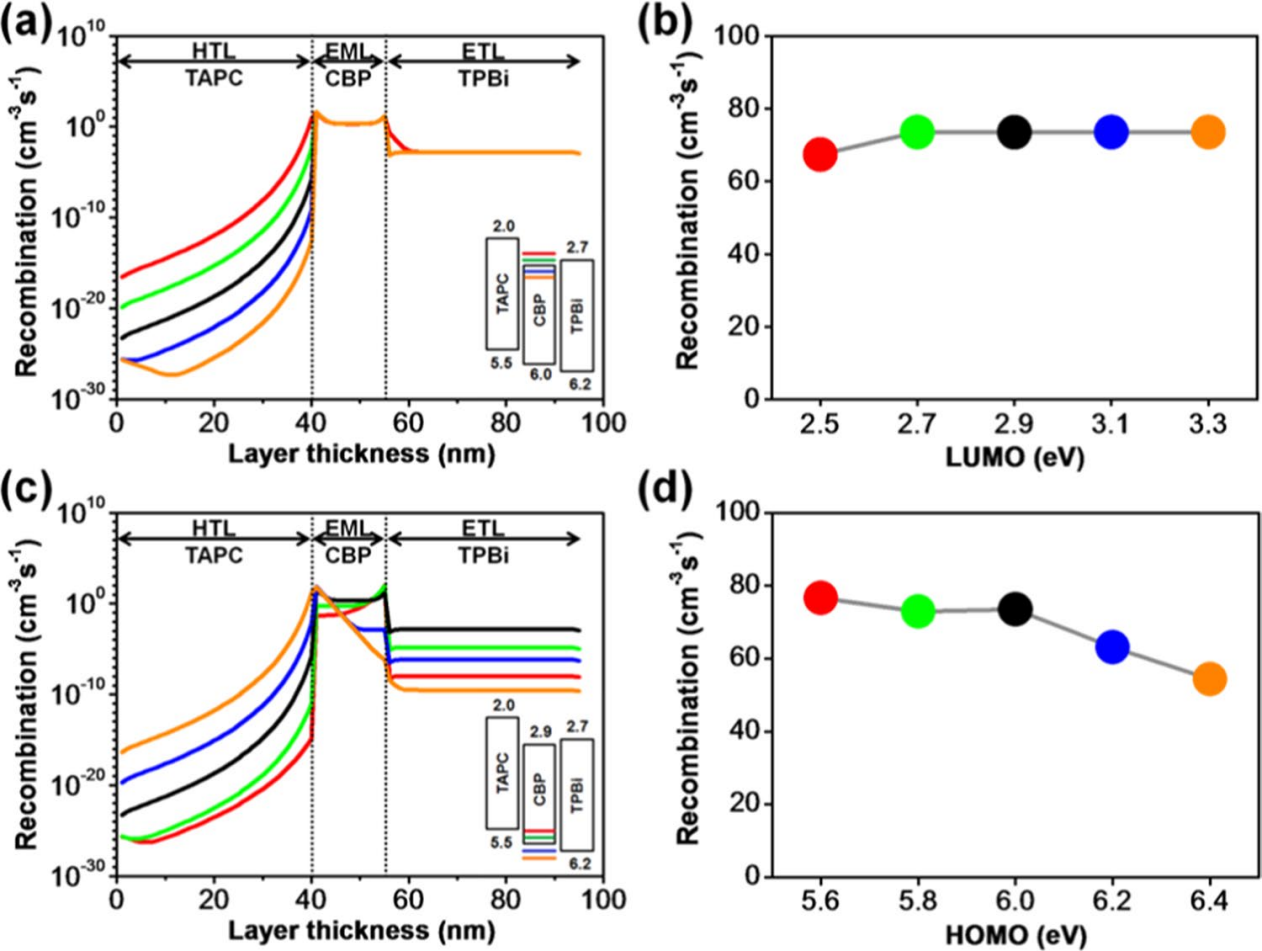
(**a**) Recombination distribution profiles in each organic layer corresponding to the simulated LUMO levels of CBP, (**b**) effects of LUMO levels of CBP on the total radiative recombination rates across the EML, (**c**) recombination distribution profiles in each organic layer corresponding to the simulated HOMO levels of CBP, and (**d**) effects of HOMO levels of CBP on the total radiative recombination rates across the EML of TPBi composing OLEDs.

The effects of the molecular orbital energy levels of the CBP host on the exciton formation, exciton diffusion and exciton quenching across the devices are expressed in Fig. 6. Figure 6a illustrates the simulation outcomes of the LUMO level variations of TAPC on the recombination rates across the device and Fig. 6b shows the total radiative recombination rates within the EML with different LUMO levels. The simulation outcome demonstrates that the recombination rate across the HTL is heavily influenced by the LUMO level of the CBP, while that of the EML and ETL shows a relatively constant value. The total radiative recombination rate across the EML is impervious (73.5 cm^−3^s^−1^), as the LUMO value varies from 3.3 to 2.7 eV. However, further increasing the LUMO level to 2.5 eV, abatement in the total radiative recombination (67.3 cm^−3^s^−1^) is observed. This decrease may result from forming an interface barrier at the EML/ETL interface. As the LUMO value increases from 2.7 to 2.5 eV, it builds an electron injection barrier at the interface resulting in misalignment of the energy levels and then causing a deficiency of electrons. Moreover, the barrier built at 2.5 eV not only affects the injection of electrons from the HTL to the EML but also accumulates excess electron in the HTL at the interface as shown in Fig. 6a. Interestingly, the total exciton formation rate across the HTL was considerably decreased from 4.1 to 1.3 × 10^−13^ cm^−3^s^−1^ as the LUMO varies from 2.5 to 3.3 eV. This phenomenon may cause from the continuous decreasing of the electron barrier at the HTL/EML interface leading to the overflow of the electrons from the EML to HTL. These results suggest that there should be an optimized energy level with an appropriate barrier/gap between the constructive layers to achieve an effective charge transport, accumulation and high exciton recombination across the device.

Figure 6c illustrates the simulation outcomes of the HOMO level effects of the CBP on recombination profiles across the organic layers and interfaces. Figure 6d shows the total radiative recombination rates within the EML with different LUMO levels. The total recombination profile across the whole device is significantly influenced by the HOMO level of CBP. The total radiative recombination rate within the EML decreased from 73.5 to 63.1 cm^−3^s^−1^, a decrement of 14%, as the HOMO value of CBP was tuned from 6.0 to 6.2 eV. The associated reason behind this may be enlarging the energy barrier between the HTL and EML as well as shrinking the difference between the EML and ETL, which leads to a weaker hole transport from the HTL to EML followed by intemperate hole transfer from the EML to ETL. By further decreasing the HOMO value to 6.4 eV, the total radiative recombination rate continued to reduce to 54.3 cm^−3^s^−1^. This may be due to fewer exciton formations within the emissive layer, because of (i) too large hole injection barrier between the HTL and ETL, i.e. 0.9 eV, and (ii) overflow of holes at the EML/ETL interface. In contrast, the total radiative recombination rate within the EML is slightly increased from 73.5 to 76.6 cm^−3^s^−1^ as the HOMO value increased from 6.0 to 5.6 eV. It is because of that there is no HOMO barrier, as the case of 5.6 eV, between the HTL and EML enabling more effective transfer of holes to the emissive layer hence triggering more excitons within the EML causing a more efficient energy transfer between host-guest system. Meanwhile, the ETL/ETL interface also acts as a more efficient hole blocking barrier enhancing the formation of excitons in the EML. It is worth to note that the total recombination within the HTL exponentially increases from 9.8 × 10^−16^ to 7.1 cm^−3^s^−1^ as the HOMO varies from 5.6 to 6.4 eV. The highest recombination peak at the EML/ETL interface successively decreases from 1.1 × 10^2^ to 6.3 × 10^−7^ cm^−3^s^−1^, while it increases from 4.5 × 10^−2^ to 64.1 cm^−3^s^−1^ at the HTL/EML interface with a decrease in HOMO values. This result implies that a reasonable cascade structure can effectively enhance the transfer and confinement of the carriers.

#### Effect of electron transporting layer

**Figure 7:**
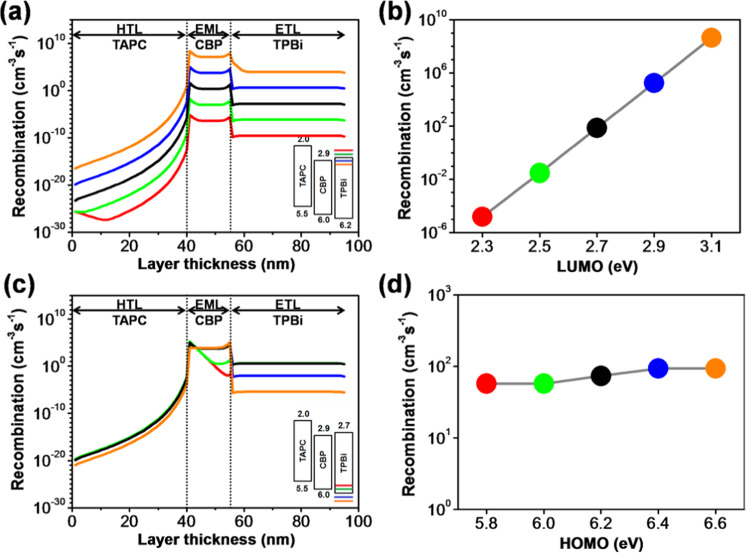
(**a**) Recombination distribution profiles in each organic layer corresponding to the simulated LUMO levels of TPBi, (**b**) effects of LUMO levels of TPBi on the total radiative recombination rates across the EML, (**c**) recombination distribution profiles in each organic layer corresponding to the simulated HOMO levels of TPBi, and (**d**) effects of HOMO levels of TPBi on the total radiative recombination rates across the EML of TPBi composing OLEDs.

The molecular-orbital alignment and carrier mobility of an electron transport material play a key and integrated role in considering a highly-efficient and long-lifespan OLED device^41,42^. Figure 7 reveals the simulation outcome of the molecular orbital energy level effects of TPBi on the exciton generation across the layers and interfaces. The simulation outcome demonstrates that the exciton recombination rate within the device is heavily influenced by the LUMO level of the ETL. With a decrease in the LUMO value of the TPBi, the recombination profile shifts up significantly across the whole device indicating the vital role of the ETL as shown in Fig. 7a. The total radiative recombination rate within the emissive layer exponentially increases from 1.2 × 10^−5^ to 73.5 cm^−3^s^−1^ as the LUMO value decreases from 2.3 to 2.7 eV. The recombination rate further increases to 4.2 × 10^8^ cm^−3^s^−1^ as the LUMO value continually decreases to 3.1 eV, as shown in Fig. 7b. The reason behind this may attribute to the enhancement of electron injection causing from reducing the entrance barrier from the cathode (4.3 eV) to ETL. Generally speaking, electrons are the minor carriers in the device, and their behaviors dominate the device performance. Reducing the barrier of the cathode/ETL interface can profoundly enhance the injection of electrons and then promote the formation of excitons and hence increasing the recombination across the whole device. That is why the device harvests exponential-higher recombination rates as the LUMO level of TPBi continually decreases from 2.3 to 3.1 eV.

Figure 7c illustrates the simulation outcomes of the HOMO level effects of the TPBi on recombination profiles across the organic layers and interfaces. Figure 7d shows the total radiative recombination rates within the EML with different LUMO levels. The total recombination profile across the whole device is influenced by the HOMO level of the TPBi. Notably, the recombination rates across the ETL were significantly persuaded by the HOMO level. The altitude of the recombination peak in the EML decreases at HTL/EML interface and increases at the interface of EML/ETL simultaneously when the HOMO level varies from 5.8 to 6.6 eV as shown in Fig. 7d. The reason for this might be due to the enhancement in hole blocking ability of the ETL, i.e., deep HOMO level restricts the migration of holes from the emissive layer interface and then enhances the accessibility of holes for radiative recombination. The radiative recombination rate across the EML shows a constant value for different HOMO levels, and it might be due to migrations of the same electrons from the ETL to EML. Within the EML, holes are generally the major carries with faster moving mobility, so only limited holes can recombine with electrons to form excitons. For high HOMO level (as the case of 5.8 eV), the excess holes may flow smoothly into the ETL resulting in high recombination in ETL. In this case, the electrons within the EML can meet the holes at the HTL/EML interface for the existing of a 0.9 eV electron barrier leading to a highest radiative recombination at the interface. For deep HOMO level (as the case of 6.6 eV), holes can be blocked at the EML/ETL interface more or less. As a result, fewer holes flow into the ETL, and radiative excitons form in the EML more evenly. That is why the recombination profile across the EML becomes smooth with two little peaks at the boundaries. It is also why the recombination in ETL significantly decreases from 1.5 × 10^2^ to 1.4 × 10^−4^ cm^−3^s^−1^ as HOMO level decreases from 5.8 to 6.6 eV. The recombination rate across the HTL decreases from 3.4 × 10^−3^ to 8.8 × 10^−5^ cm^−3^s^−1^ as HOMO level decreases from 5.8 to 6.6 eV.

### Carrier recombination distribution of the BPhen based devices

Charge carrier mobility is one of the critical parameters as determining the performance of organic electronic devices. Regarding electrons are usually the minor carries in the OLED devices, injection and transportation of them would dominate the device performance. It is necessary to clarify the degree of influence and importance of the electron mobility of an ETL. Basing on these inspirations and notions, we replace the electron transporting material from TPBi to Bphen to appraise the recombination rate dynamics across the devices, Bphen possessing higher electron mobility (µ_e_ = 5.2 × 10^−4^ cm^2^V^−1^s^−1^) than the TPBi (µ_e_ = 3.3 × 10^−5^cm^2^V^−1^s^−1^). Moreover, comparing to TPBi, Bphen also shows better abilities on hole-blocking and electron injection for its deeper HOMO level and lower LUMO level, respectively^41,42^.

**Figure 8:**
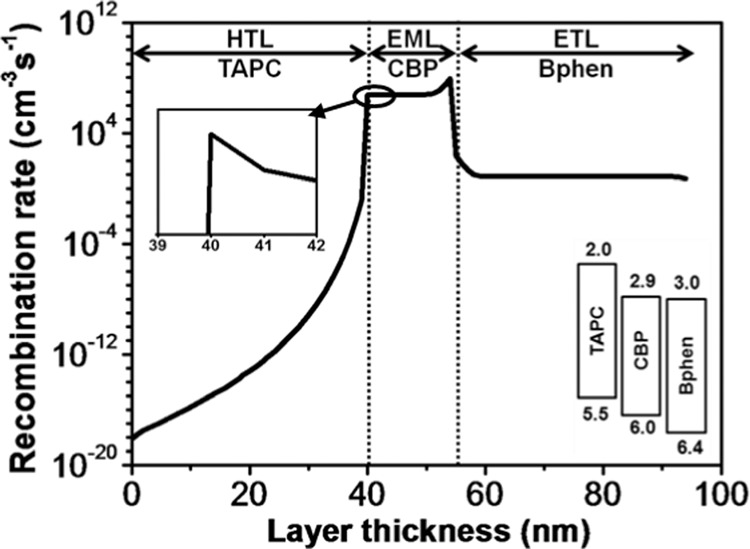
Recombination profile across the layers of the Bphen-ETL based control device.

Figure 8 shows the recombination profile of the Bphen composing control device. The simulation outcomes show that the recombination occurred in the EML is significantly higher than that of the HTL and ETL, which would lead to a highly efficient and prolonged lifespan OLED device. The expected good result might be related to several benefits causing by the use of Bphen such as efficient electron transport, high hole-blocking ability, and suitable cascade energy levels for the studied device. Notably, the total radiative recombination of the Bphen composing control device (1.4 × 10^8^ cm^−3^s^−1^) is ~10^7^ times higher than that of the TPBi one which could guarantee a much better performance of the Bphen composing device. Again, the expected excellent performance of the Bphen composing device should attribute to the mentioned benefits via the use of Bphen. Moreover, the highest recombination peak observed at the EML/ETL interface (8.8 × 10^8^ cm^−3^s^−1^) is >100 times higher than that of the HTL/EML interface (5.9 × 10^6^cm^−3^s^−1^) which is shown as the inset in Fig. 8.

The reasons that the magnitude and maximum peak site of the recombination profiles are different for the Bphen (Fig. 8) and TPBi (Fig. 4) composing control devices may be due to the differences in energy level structures as well as the carrier mobility. For Bphen control device, it possesses a 0.4 eV hole blocking barrier at the EML/ETL interface. However, that of TPBi device is only 0.2 eV. The higher EML/ETL interface barrier of the Bphen device indicates that it could block more holes at its EML/ETL interface, which means the more hole accumulations at the interface. Besides, the comparatively high electron mobility and low electron injection barrier from the cathode (LiF/Al) to ETL in Bphen control device that helps an effective injection and transportation of electrons which can be evidenced from the relatively high amount of recombination across the ETL. These two factors are responsible for why the Bphen control device shows a maximum recombination peak at the EML/ETL interface in the EML region. Most of the electrons are consumed at the EML/ETL interface, while HTL/EML interface can block few successively transported and through-passed electrons regardless of its extreme high electron blocking barrier, i.e. 0.9 eV for Bphen device. That is why Bphen composed control device shows an inconspicuous recombination peak at the HTL/EML interface shown as the inset of Fig. 8. In contrast, the comparatively low hole blocking at the EML/ETL interface, the high enough electron barrier at the ETL/cathode interface, and the low electron mobility of the TPBi control device results in two-peaked character combination profile with a low recombination rate. The simulation outcomes are well matched with our previous published experimental ones, i.e., Device V and VI^22^.

#### Effect of hole transporting layer

**Figure 9:**
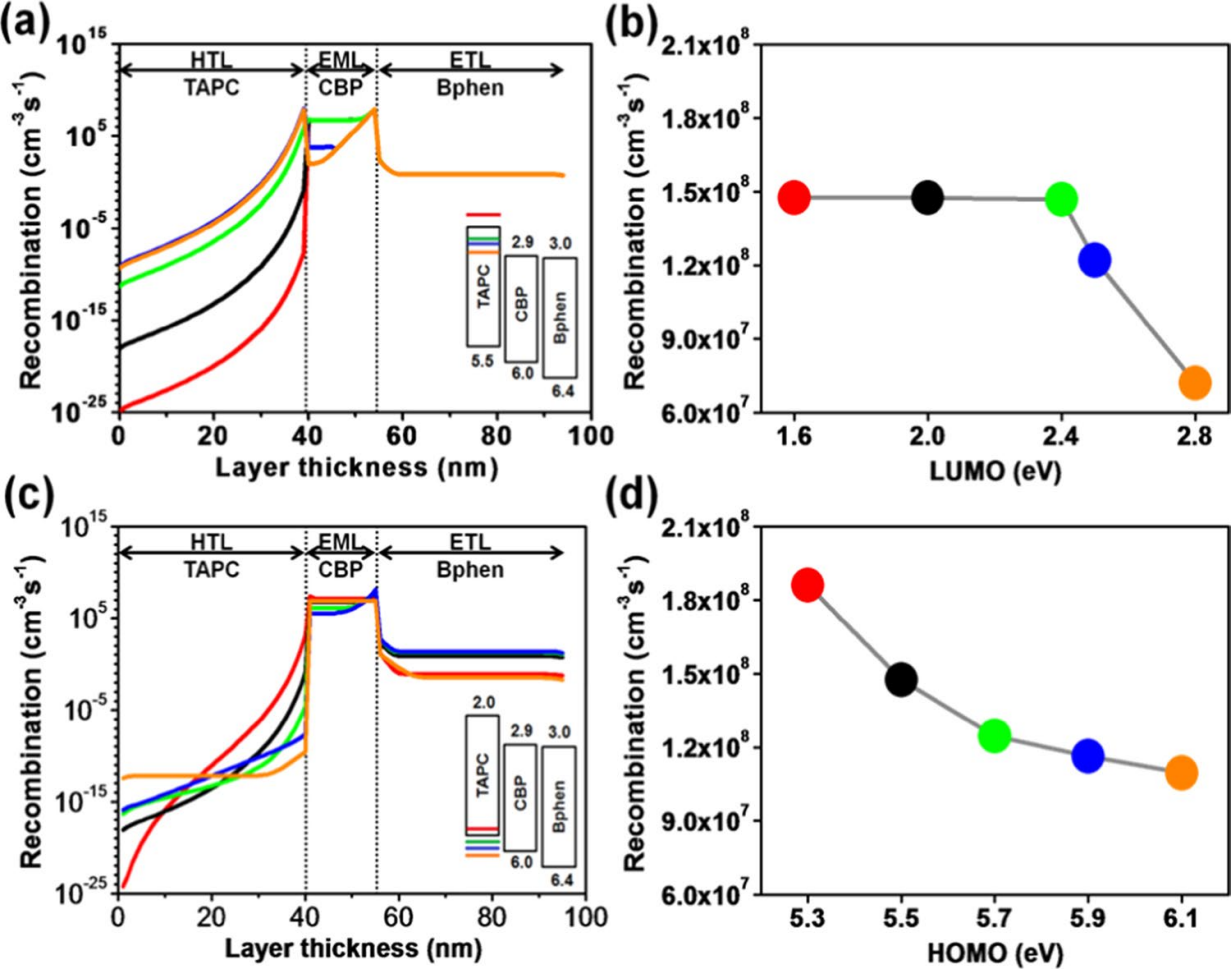
(**a**) Recombination distribution profiles in each organic layer corresponding to the simulated LUMO levels of TAPC, (**b**) effects of LUMO levels of TAPC on the total radiative recombination rates across the EML, (**c**) recombination distribution profiles in each organic layer corresponding to the simulated HOMO levels of TAPC, and (**d**) effects of HOMO levels of TAPC on the total radiative recombination rates across the EML of Bphen composing OLEDs.

Figure 9 reveals the effect of the molecular orbital energy levels of TAPC on the recombination profiles across the organic layers and interfaces. Figure 9a illustrates the influence of the LUMO levels of the TAPC HTL on the recombination rates across the device, and Fig. 9b shows the total radiative recombination rates within the EML with different LUMO levels. It concludes that the LUMO level shows different influences on the recombination trends within the HTL and EML. As the LUMO level changes from 1.6 to 2.8 eV, the recombination profile across HTL monotonously increases which could contribute to the loss of electron blocking ability of TAPC resulting in the promotion of electron migration from the EML to the HTL.

As regarding the EML, the total radiative recombination across the EML is almost insusceptible to LUMO level ranging from 1.6 to 2.4 eV, i.e., from 1.47 × 10^8^ to 1.46 × 10^8^ cm^−3^s^−1^. Then, the total radiative recombination rate within the EML decreases from 1.46 × 10^8^ to 1.22 × 10^8^ cm^−3^s^−1^, as the LUMO level of TAPC is tuned from 2.4 to 2.5 eV. The total radiative recombination rate within the EML further decreases to 7.2 × 10^7^ cm^−3^s^−1^, as the LUMO level of TAPC is set to 2.8 eV. The non-decreasing of the total radiative recombination, i.e., from 1.6 to 2.4 eV, may attribute to the excellent hole transport and injection of the TAPC HTL which provides enough holes to compensate the overflowing electrons from the EML to HTL and also to afford substantial holes to trigger the radiative recombination in the EML. However, at 2.5 eV LUMO level, the balanced injection of the holes and electrons in the EML is broken for the 0.4 eV energy barrier at the HTL/EML interface is not enough to impede sufficient electrons within the EML. That is why at this point the total radiative recombination starts to decline. As the LUMO level is further set to 2.8 eV, the energy barrier is now only 0.1 eV, which is too small to block the electrons, resulting in too extra electrons flowing into the HTL causing the non-radiative recombination in the HTL that usually leads to poor device performance and lifetime^16^. It is also worth to note that the recombination profiles of 2.5 and 2.8 eV are different from others, as shown in Fig. 9a. Both of them possess only one peak within the EML, i.e., at the EML/ETL interface. Instead of showing a second peak at the HTL/EML interface, they show depletion of radiative recombination at the interface. However, they possess a more visible second peak in the HTL at HTL/EML interface. The reason may again attribute to the loss of electron blocking ability at the HTL/EML interface which leads to extra electron overflows from EML to HTL resulting in the depletion of recombination in the EML and formation of the second peak in the HTL. Moreover, the successively transported holes, i.e., from the HTL to EML, move toward the ETL and then accumulate at the hole blocking EML/ETL interface for possessing higher mobility than that of electrons. It explains why the recombination profile only has a maximum peak at the EML/ETL interface within the EML in the cases of 2.5 and 2.8 eV TAPC.

**Figure 10:**
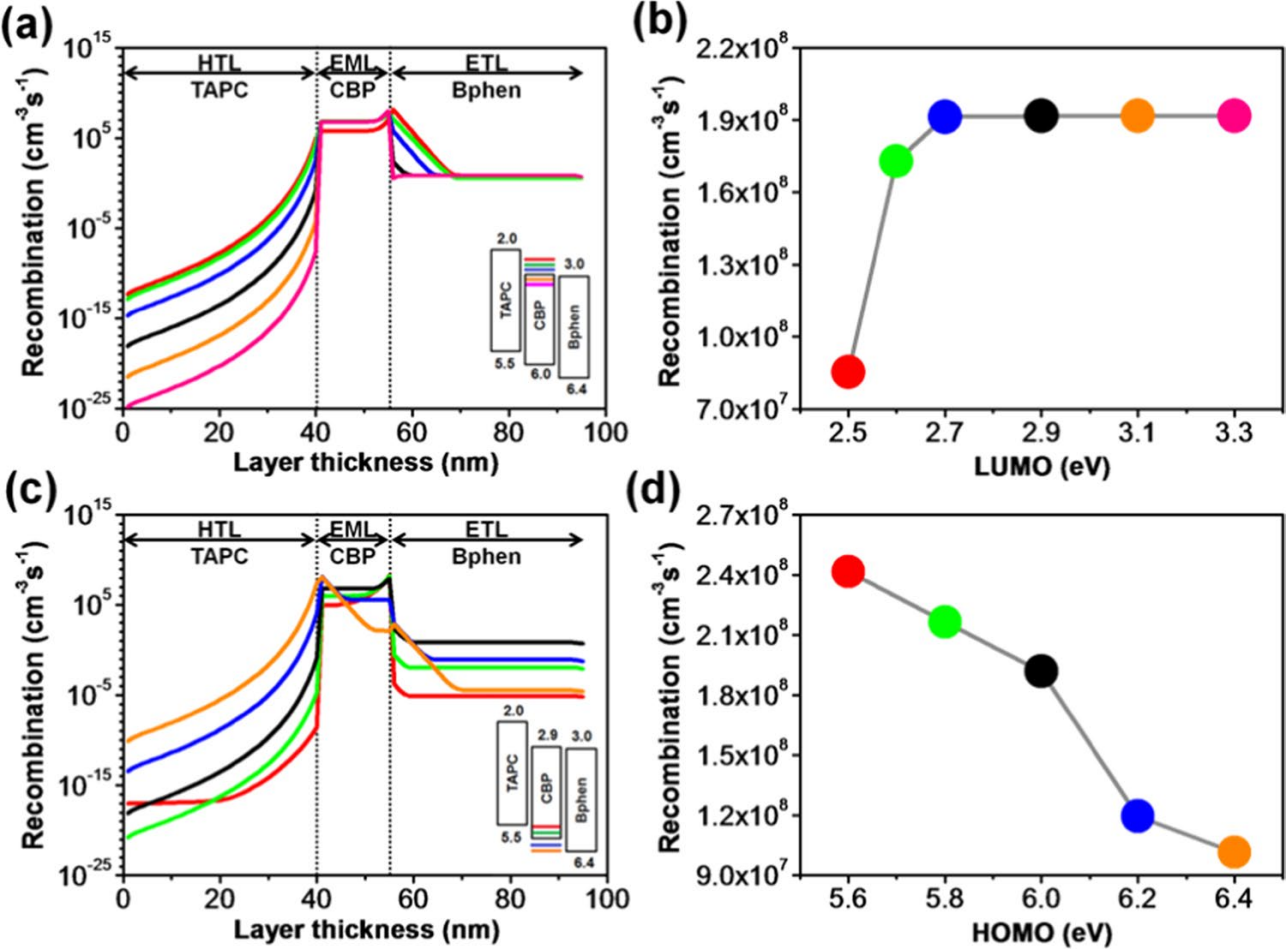
(**a**) Recombination distribution profiles in each organic layer corresponding to the simulated LUMO levels of CBP, (**b**) effects of LUMO levels of CBP on the total radiative recombination rates across the EML, (**c**) recombination distribution profiles in each organic layer corresponding to the simulated HOMO levels of CBP, and (**d**) effects of HOMO levels of CBP on the total radiative recombination rates across the EML of Bphen composing OLEDs.

Figure 9c illustrates the influence of the HOMO levels of TAPC on the recombination rates across the device and Fig. 9d shows the total radiative recombination rates within the EML with different HOMO levels. As shown in Fig. 9d, the total radiative recombination rate within the EML decreases from 1.8 × 10^8^ to 1.1 × 10^8^ cm^−3^s^−1^, as the HOMO level changes from 5.3 to 6.1 eV. The reason behind this should be due to the increase in hole injection difficulty caused by the change of TAPC HOMO level. Although decreasing the TAPC HOMO level can reduce the HTL/EML energy gap to facilitate the injection of holes at this interface, it also increases the energy gap between the ITO and HTL to impede the injection of holes. As a result, not only the recombination rate within the EML is decreasing as the TAPC HOMO level becomes deep, but also that within the HTL is declining, as shown in Fig. 9c. It is worth to point out the profile shape within the EML changes with the TPAC HOMO level as well. When the HOMO level is set as 5.3 eV, i.e., the red color one, the profile possesses a maximum peak at the HTL/EML interface. While the TAPC HOMO decreases to 5.9 eV, the concentrated recombination zone shifts toward the EML/ETL and then shows a maximum peak at the EML/ETL interface, i.e., the blue one. Further modulating the HOMO level to 6.1 eV, the recombination rate becomes evenly without no visible peak in the EML, i.e., the orange curve one drawn in Fig. 10c. There is no energy barrier at the HTL/EML interface at 5.9 eV TAPC HOMO level. Instead, it forms an energy well in the EML. Therefore, the holes overcome the ITO/HTL energy barrier can also go through the EML and jump into the Bphen ETL easily. It is why at 5.9 eV the recombination in the EML shows a plateau profile without any peak. Notably, the recombination in the ETL is first increasing in 5.3 to 5.9 eV TAPC HOMO level and then declining at 6.1 eV energy level. The increment of the recombination may be a consequent result of the accumulation of holes at the EML/ETL interface to promote the injection of holes from the EML to ETL due to the peak shift of the recombination in the EML caused by the changing of the HOMO energy level. However, for the 6.1 eV, there is no accumulation of holes at the EML/ETL interface in the EML to induce the hole transfer from the EML to ETL leading to the decrement of recombination in the ETL. Moreover, the different behaviors between 5.9 and 6.1 eV ones indicate that 0.4 eV of the energy gap could be regarded as an effective barrier.

#### Effect of host

Figure 10 reveals the simulation outcome of the molecular orbital energy level effects of CBP on the exciton generation across the layers and interfaces. The simulation outcome demonstrates that the exciton recombination rate within the device is heavily influenced by the LUMO and HOMO levels of the host CBP. Figure 10a illustrates the simulation outputs of the LUMO level effects of CBP on recombination profiles across the organic layers and interfaces. Figure 10b shows the total radiative recombination rates within the EML with different LUMO levels. As shown in Fig. 10a, the recombination rate within the HTL exponentially increases with the change of CBP LUMO level from 3.3 to 2.5 eV. The reason may be due to the loss of electron blocking potential at the HTL/EML interface. The change of CBP energy level also alters the electron barrier gap between the EML and ETL, resulting in the different recombination profile in the ETL. For the cases of 3.3 and 3.1 eV energy level, there is an electron trap at the EML/ETL interface, which means the electrons can cross the EML/ETL interface smoothly. It is why these two profiles are overlapping within the ETL accompanying with no electron accumulation at the EML/ETL interface. From the 2.9 to 2.5 eV ones, the electron barrier was increased with the rise of energy level, increasing the difficulty of electron transfer from the ETL to EML. As a result, the total recombination rate in the ETL increases from 2.5 × 10^2^ to 1.1 × 10^8^ cm^−3^s^−1^ as LUMO changes from 3.3 to 2.5 eV. Moreover, it is worth to point out that the amount of electron accumulation within the ETL is increased as the interface barrier is enlarged, i.e., from 2.9 to 2.5 eV.

Unlike showing a simple trend in the HTL and ETL, the trend of recombination rate within the EML shows a two-stage-profile instead. The first stage, the total radiative recombination across the EML is almost insusceptible, i.e., around 1.9 × 10^8^ cm^−3^s^−1^, to the LUMO level at the range between 3.3 to 2.7 eV. However, as the LUMO level is set to 2.6 eV, a curtailment in the recombination rate (1.7 × 10^8^ cm^−3^s^−1^) is recognized, which is the start of the second stage. The radiative exciton formation across the EML significantly drops to 8.5 × 10^7^ cm^−3^s^−1^ as the LUMO level is further raised to 2.5 eV. The unchanged total radiative recombination in the interval of 3.3 to 2.7 eV could attribute to two conflict factors, i.e., the loss of electron blocking at the HTL/EML interface against the use of Bphen. As mentioned above, the comparative low injection barrier at ETL/cathode as well as the high electron mobility of Bphen could guarantee to supply substantial electrons from the cathode to ETL. For 3.3 and 3.1 eV ones, although the electron trap at the EML/ETL interface could promote the electron injections from the ETL to EML, the loss of electron blocking at the HTL/EML interface could lead to overflowing of excess electrons from the EML to HTL. For the cases of 2.9 and 2.7 eV, the electron barrier at the EML/ETL interface might prevent the injection of electrons, but the substantial supplied electrons pile up at the interface to inject more electrons. As a result, at the span of 3.3 to 2.7 eV, the promoted injected electrons, i.e., form the ETL to EML, is compensated by the overflowing of electrons causing from the loss of electron blocking at the HTL/EML interface to maintain the same recombination profile and rate in the EML. However, as the LUMO level is set to 2.6 eV, the pileup-promoted electron injection mechanism no longer compensates the overflow of electrons which indicates that the available electrons in the EML are reduced. It is why the total radiative recombination across the EML starts to decline at 2.6 eV. For the case of 2.5 eV, the loss of electrons and the barrier at the EML/ETL are too large to compensate for, causing a dramatical drop in the total radiative recombination. Two more things are worth to note. First, the twist of recombination trend at 2.6 eV indicates that 0.4 eV energy gap at the EML/ETL interface is demanded to prevent/block the transfer of electrons. Second, not only the total radiative recombination is insusceptible at the range between 3.3 to 2.7 eV, but also the efficient recombination fraction, i.e., the ratio of total radiative recombination to overall recombination across the device, remains a high score indicating a good device performance. The efficient recombination fractions are 98%, 98%, 98%, 98%, 88%, and 43% for the LUMO level of 3.3, 3.1, 2.9, 2.7, 2.6, and 2.5 eV, respectively.

Figure 10c describes the HOMO level effects of CBP on the recombination rates across the device and Fig. 10d demonstrates the total radiative recombination rates within the EML with different HOMO levels. As shown in Fig. 10c, the recombination rate within the HTL raises with the drop of CBP HOMO level for increasing the hole barrier gap at the HTL/EML interface. The drop of CBP HOMO level also alters the recombination trend within the ETL. From 5.6 to 6.0 eV, the drop of energy level increases the recombination rate within the ETL. However, further decreasing the energy level, i.e., the cases of 6.2 and 6.4 eV, the recombination rate declines. The reason is discussed below. As a hole is delivered from the HTL to ETL, it needs to overcome two energy barriers, i.e., HTL/EML and EML/ETL interfaces. These two gaps vary with the CBP HOMO level in the opposite trend. The gap at the HTL/EML increases with the drop of CBP HOMO level, but that of EML/ETL decreases instead. Usually, the larger gap one could dominate the hole transfer behavior. As a result, the total amount of delivered holes in the ETL increases with the drop of energy level at the interval of 5.6 to 6.0 eV for decreasing the dominated EML/ETL gap to facilitate the transfer of holes. For the cases of 6.2 and 6.4 eV, the HTL/EML gap becomes the bottleneck to constrain the transfer of holes from HTL to EML, resulting in the decrease of hole amounts in the ETL.

In the EML region, distinguishable profiles can be recognized by identifying the maximum recombination peak position. For the cases of 5.6, 5.8 and 6.0 eV energy level, the maximum recombination peaks are near to EML/ETL interface. However, for 6.2 and 6.4 eV ones, the maximum peak shifts to the HTL/EML interface. The reason of forming a maximum peak close to the EML/ETL interface for the former group would attribute to enough hole blocking potential as well as high electron mobility of the Bphen as discussed in the part of Bphen composing control device. For the latter group, the hole barrier at the HTL/EML interface becomes too giant to transfer the holes from the HTL to EML effectively. Meanwhile, the existed 0.9 eV electron barrier at the HTL/EML interface instead blocks most passed electrons. That is why the latter group shows the maximum recombination peak at the HTL/EML interface. It is worth to point out the high total radiative recombination of 5.6 and 5.8 eV ones result from the concentrated exciton formation occurred at the EML/ETL interface. In contrast, the profile of 6.0 eV one shows an overall high and even recombination within the EML with a comparatively small peak at the EML/ETL interface. Moreover, the efficient recombination fractions are 99%, 99%, 98%, 63%, and 54% for the HOMO level of 5.6, 5.8, 6.0, 6.2, and 6.4 eV, respectively. The high efficient recombination fraction of 6.0 eV one as well as its even recombination profile within the EML indicates that it may yield good device characteristics.

#### Effect of electron transporting layer

**Figure 11:**
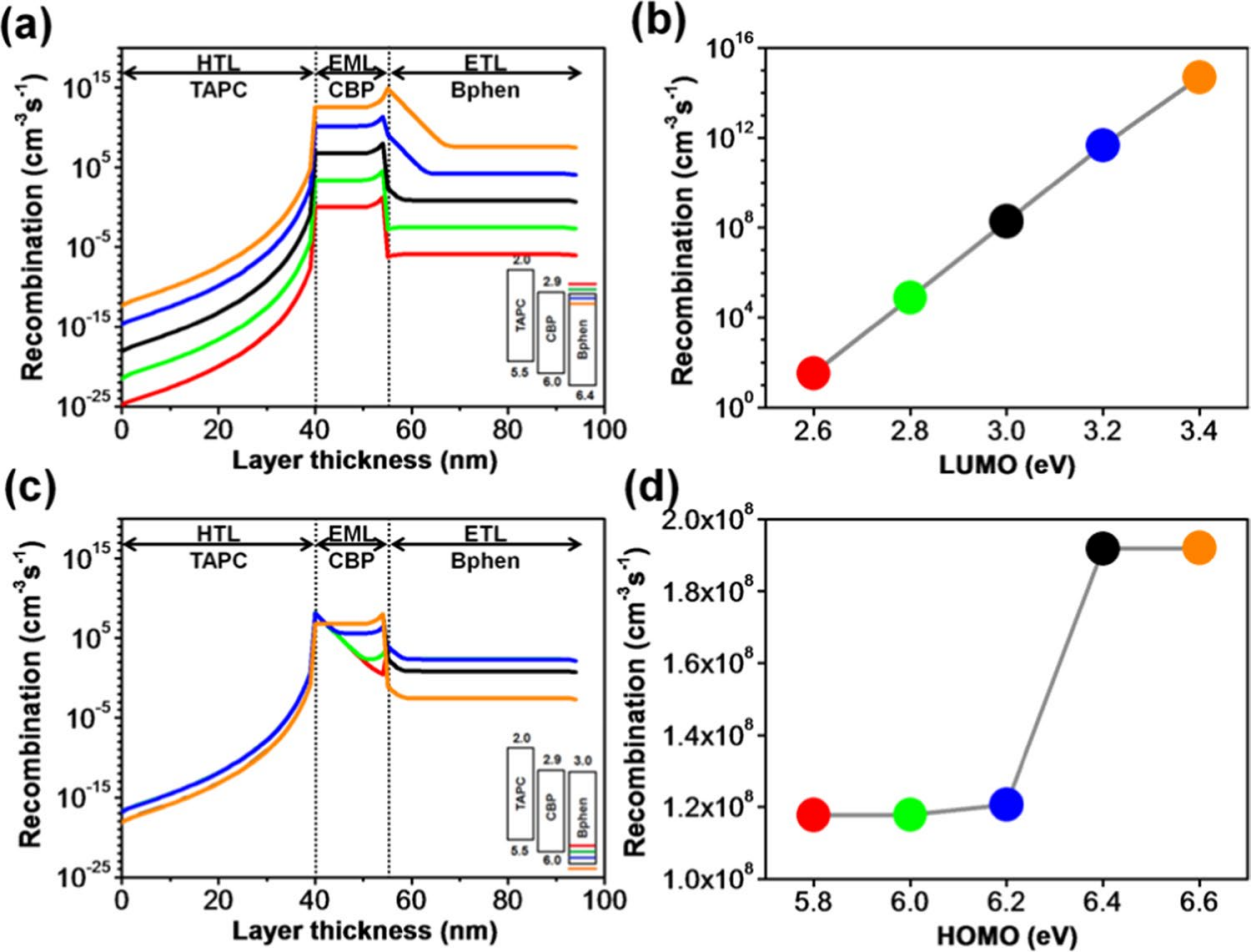
(**a**) Recombination distribution profiles in each organic layer corresponding to the simulated LUMO levels of Bphen, (**b**) effects of LUMO levels of Bphen on the total radiative recombination rates across the EML, (**c**) recombination distribution profiles in each organic layer corresponding to the simulated HOMO levels of Bphen, and (**d**) effects of HOMO levels of Bphen on the total radiative recombination rates across the EML of Bphen composing OLEDs.

Figure 11 demonstrates the recombination rate profiles across the organic layers influenced by the changes of Bphen LUMO levels. Figure 11a shows the exciton recombination profiles within EML corresponding to the ETL LUMO levels. Figure 11b shows the total radiative recombination rates within the EML with different ETL LUMO levels. As shown in Fig. 11a,b, the recombination profile and radiative recombination rate exponentially increase with the decrease of LUMO level, i.e., from 2.6 to 3.4 eV, causing from the decrease of the electron barrier at the ELT/cathode interface. It is interesting to note that the efficient recombination fraction is constant for the LUMO levels 2.6, 2.8, 3.0, and 3.2 eV, which is 98%. The high efficient recombination fraction, as well as high total radiative recombination rate, indicates these devices may possess superior device performance. However, the efficient recombination fraction drops to 43% as the LUMO is set to 3.4 eV, which may lead to poor device performance. Moreover, the electron pileup profiles are also observed at the EML/ETL interface for the existence of electron injection barrier for the cases of 3.2 and 3.4 eV, which again facilitates the electron injection from the ETL to EML to yield a high total radiative recombination rate.

Figure 11c describes the HOMO level effects of Bphen on the recombination rates across the device and Fig. 11d demonstrates the total radiative recombination rates within the EML with different HOMO levels. Regardless of the insensitive of the total radiative recombination rate with a shape of step to the HOMO level of the BPhen, the recombination profile within the EML is sensitive to the energy level as expressed in Fig. 11c,d. Also, the recombination profile peak position shifting with the HOMO levels is observed. For the cases from 5.8 to 6.2 eV HOMO level, holes can jump to Bphen ETL easily for the low EML/ETL hole barrier resulting in insufficient hole blocking at the EML/ETL interface. That is why from 5.8 to 6.2 eV, the total radiative recombination rate shows little increase, i.e., from 1.1 × 10^8^ to 1.2 × 10^8^ cm^−3^s^−1^. The insufficient hole blocking at the EML/ETL interface also leads to the overflow of holes from the EML to ETL. In contrast, the electrons are well blocked at the HTL/ETL interface for the existence of 0.9 eV electron barrier gap.

As the case of 5.8 eV Bphen HOMO level, the 0.2 eV hole energy trap at the EML/ETL interface leads the overflows of the hole at the interface. In contrast, the 0.9 eV barrier gap at the HTL/ETL interface can effectively block the electrons to remain in the EML with an electron pileup at the HTL/EML interface. As a result, the excitons pile up at the HTL/EML interface to form a recombination peak with an unevenly distributed profile across the EML. As the HOMO level decreases to 6.2 eV, an insufficient hole blocking barrier is formed at the EML/ETL interface. It is why its recombination profile across the EML becomes more even than that of 5.8 and 6.0 eV ones, shown as the blue curve in Fig. 11c. The lack of hole blocking of 5.8, 6.0, and 6.2 eV ones is also responsible for high recombination rate in the ETL region, which is resulting from the overflow of holes from the EML to ETL. Further decreasing the Bphen HOMO level to 6.4 and 6.6 eV, the holes can be well blocked within the EML. Hence, the profiles across the EML of them are even with a small peak at the EML/ETL interface. The good hole blocking of 6.4 and 6.6 eV ones also contribute to their low recombination rates in the ETL, which are 10 and 10^−5^, respectively. Moreover, the efficient recombination fractions are 62%, 62%, 64%, 98%, and 98% for the HOMO level of 5.8, 6.0, 6.2, 6.4, and 6.6 eV, respectively. Again, the even radiative recombination profile, as well as the high efficient recombination fraction, implies the demand of 0.4 eV gap to block the carriers.

**Figure 12:**
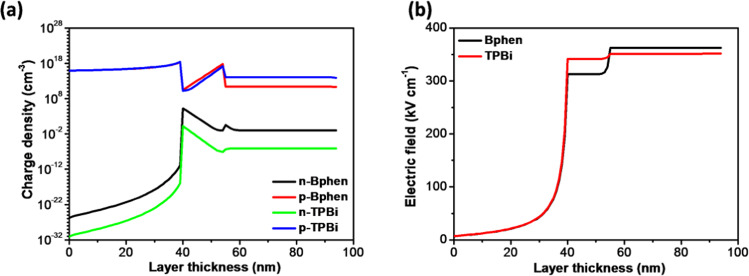
(**a**) Charge density and (**b**) electric field profile across both the Bphen and TPBi composing control devices.

Furthermore, we studied the charge density and electric field profiles for both TPBi and Bphen composing control devices represented in Fig. 12(a,b), respectively. The charge density profiles are well interrelated with the recombination profiles of the corresponding devices. For the TPBi one, the density amount gap between two carriers is large, which indicates the poor device performance being consistent with Fig. 4. In the Bphen composing device, the hole accumulation at the EML/ETL interface is high due to its deep HOMO level (6.4 eV) acting as an effective hole blocking layer, which significantly results in maximum recombination at the specific interface. Also, the electron density in the Bphen control device is considerably high because of its high electron mobility leading to a high recombination rate. A 341.7 kVcm^−1^ and 351.2 kVcm^−1^ electric field across the EML and ETL, respectively, have been observed in the case of the TPBi composing device. However, a 312.6 kVcm^−1^ and 362.3 kVcm^−1^ electric field across the EML and ETL, respectively, noted for the Bphen one. The high electric field behavior in the EML profoundly influences the device performance, and hence the recombination rate. The plausible reasons for the high electric field might be an improper charge transfer, charge carrier deficiency, or charge accumulation, which might lead to the breakdown of the dielectric strength of the layer^46^. The low electric field in EML of the Bphen composing device shows an effective charge transfer and migration across the successive layers due to its superior properties, as discussed. The electric field in the ETL of the Bphen device slightly increased that might due to the accumulation of hole at the EML/ETL interface for its deeper HOMO level leading to the better hole blocking ability that of the TPBi one^47–51^.

## Conclusion

In this study, we have demonstrated a comprehensive drift-diffusion simulation to illustrate the effect of molecular orbital energy levels of the organic materials on recombination rate in OLED devices. It is the first time that a vivid picture of recombination profiles of all of the composing organic materials is investigated, simultaneously. The outcomes reveal that exciton recombination is highly sensitive to the energy level alignment, injection barriers and charge mobilities. A low energy barrier (<0.4 eV) between the layers is the key to yield high exciton recombination. The lowest unoccupied molecular orbital (LUMO) levels of the organic layers play a more pivotal role in governing the recombination dynamics than the highest occupied molecular orbital (HOMO) levels. This study made possible to understand carrier/charge migration, transfer and exciton recombination yield within the organic transporting layers. This improved understanding will help domain experts and researchers to carry out extensive work towards effective molecular design and device engineering in organic semiconductors.
